# TGA2 signaling in response to reactive electrophile species is not dependent on cysteine modification of TGA2

**DOI:** 10.1371/journal.pone.0195398

**Published:** 2018-04-02

**Authors:** Simone Findling, Henrik U. Stotz, Maria Zoeller, Markus Krischke, Mark Zander, Christiane Gatz, Susanne Berger, Martin J. Mueller

**Affiliations:** 1 Julius-von-Sachs-Institute of Biosciences, Biocenter, Pharmaceutical Biology, University of Wuerzburg, Wuerzburg, Germany; 2 Albrecht-von-Haller Institute for Plant Sciences, Georg-August-University of Goettingen, Goettingen, Germany; University of Nebraska-Lincoln, UNITED STATES

## Abstract

Reactive electrophile species (RES), including prostaglandins, phytoprostanes and 12-oxo phytodienoic acid (OPDA), activate detoxification responses in plants and animals. However, the pathways leading to the activation of defense reactions related to abiotic or biotic stress as a function of RES formation, accumulation or treatment are poorly understood in plants. Here, the thiol-modification of proteins, including the RES-activated basic region/leucine zipper transcription factor TGA2, was studied. TGA2 contains a single cysteine residue (Cys186) that was covalently modified by reactive cyclopentenones but not required for induction of detoxification genes in response to OPDA or prostaglandin A_1_. Activation of the glutathione-S-transferase 6 (*GST6*) promoter was responsive to cyclopentenones but not to unreactive cyclopentanones, including jasmonic acid suggesting that thiol reactivity of RES is important to activate the TGA2-dependent signaling pathway resulting in *GST6* activation We show that RES modify thiols in numerous proteins *in vivo*, however, thiol reactivity alone appears not to be sufficient for biological activity as demonstrated by the failure of several membrane permeable thiol reactive reagents to activate the *GST6* promoter.

## Introduction

Oxylipins are compounds which affect growth, development and stress responses in plants [1]. Oxylipins which contain an α,ß-unsaturated carbonyl structure are reactive electrophile species, called RES-oxylipins. The biological activities of RES-oxylipins show some overlap but mostly differ from the activities of non-RES-oxylipins such as jasmonic acid (JA) and its derivatives. The RES-oxylipins 12-oxo-phytodienoic acid (OPDA) and phytoprostanes induce secondary metabolism and inhibit cell division and root growth, effects shared with JA [2].

Differences between the activities of RES and non-RES-oxylipins are obvious at the level of gene expression: only 11% of genes induced by A_1_-phytoprostanes are also induced by JA/MeJA [2]. This indicates the existence of different signal transduction pathways mediating the effects of JA derivatives on one hand and RES-oxylipins on the other hand. For JA, the signal transduction cascade has been intensively investigated. However, gene induction by OPDA or phytoprostanes is mostly regulated independently of the jasmonoyl-isoleucine (JA-Ile) receptor CORONATINE INSENSITIVE 1 (COI1) and the transcription factor MYC2 [3, 4]. The cyclophilin 20–3 protein has been identified as an OPDA binding protein [5]. However, the *cyp20-3* mutant exhibits only partial insensitivity indicating that this protein is only involved in part of the responses to RES-oxylipins. Up-regulation of gene expression, especially of genes related to detoxification, in response to RES-oxylipins was found to be dependent on the class II TGA transcription factors TGA2, TGA5 and TGA6 [2]. TGA2, TGA5 and TGA6 display partial redundancy: detoxification genes like *CYP81D11* and *GST25*, which are induced after RES and reactive oxygen species (ROS) stress, are regulated by different combinations of TGA factors [3]. Class II TGA transcription factors are also essential for establishment of JA/ethylene- and salicylic acid-dependent defense responses as well as systemic acquired resistance (SAR) [6, 7] as well as for induction of detoxification genes upon xenobiotic stress [8, 9]. To date, it is not clear how the different functions of class II TGA transcription factors are controlled.

Transcription factors are often regulated at the posttranslational level which allows rapid control of protein activity. RES-oxylipins can form covalent adducts with nucleophiles such as thiol and amino groups in peptides and proteins [10, 11]. Thus, covalent modification of proteins by RES-oxylipins might be a possible signaling mechanism. In fact, in animals covalent modification of specific proteins by prostaglandins, C20-RES-oxylipins in animals, has been described [12, 13]. Protein lipidation alters the activity of e.g. the transcription factor NfkappaB [14] and the mammalian thioredoxin reductase TrxR [12].

In animals, RES primarily modify target proteins at cysteine residues [15]. Also in plants, formation of RES-oxylipin carbonyls and subsequent protein modification occurs [16]. Especially for prostaglandin A_1_ (PGA_1_) modification of several proteins has been found *in vivo* [17]. This *in vivo* labeling approach, however, identified only abundant proteins. Here we determined whether, to which extent and at which amino acid residue TGA2 was covalently modified by PGA_1_ or OPDA and whether this modification was necessary for regulating the activity of TGA2 in response to RES-oxylipin treatment.

## Materials and methods

### Plant material and growth conditions

The triple knock out mutant *tga256* was described by and obtained from [6]. Overexpression lines of TGA2 in the background of the *tga256* mutant were generated and described by [7]. The C186S mutation in TGA5 was achieved by generating two PCR products on pDONR201/TGA2 using primer pairs (TCGCGTTAACGCTAGCATGGATCTC (P1) & GAGCCACAAGAAAGATCTCTCAGCTG and GTAACATCAGAGATTTTGAGACAC (P2) & CAGCTGAGAGATCTTTCTTGTGGCTC) and subsequent amplification by overlapping PCR (primers P1 and P2) followed by recombination into pB2GW7. Sequencing confirmed that the mutation had been introduced as planned. For generating transgenic plants, binary plasmids were electroporated into *Agrobacterium tumefaciens* strain GV3101 (pMP90). The resulting agrobacteria were used to transform *tga256* plants. Seeds obtained from homozygous F2 plants were used for the analysis.

For qPCR analysis, seeds were surface sterilized by treatment with 70% ethanol for 90 s and with 1.5% sodium hypochlorite and 0.1% tween for 20 min followed by five wash steps with water. Seedlings were grown in 24 well plates (8 seedlings per well) containing 1 ml of Murashige and Skoog medium with 3% (w/v) sucrose on an orbital shaker under previously described conditions [2].

The *GST6*::*luc* line has been described by [18]. For *GST6*::*luc* luminescence measurements seedlings were grown in MS medium without sucrose under conditions described above. For experiments with protein extracts Col-0 plants were grown in soil under long day conditions (16 h light, 8 h dark) for 6–8 weeks.

### Bacterial cultivation and isolation/purification of recombinant His-TGA2

His-TGA2 [8] was expressed in *E*. *coli* BL21 (DE3) containing the plasmid pAC28 [19]. pAC28 harbors the open reading frame of *TGA2* with a N-terminal 6xHis-tag sequence [19]. Recombinant TGA2 was purified by immobilized metal ion affinity chromatography (Ni-TED resign, Machery Nagel, Dueren, Germany) according to the manufacturer`s protocol. TGA2 concentration was estimated under consideration of TGA2 specific parameters (http://web.expasy.org/protparam/) by using Nano-Drop 1000 (Thermo-Scientific, Hamburg, Germany) or by using the Bradford assay with BSA as a standard [20].

### Preparation of protein extracts:

Leaf material (2 g) was shock frozen in liquid nitrogen and thoroughly ground using mortar and pestle. Homogenized plant material was then transferred to 15 ml of sodium phosphate buffer pH 7 containing 2 to 4 mM DTT (Sigma, Taufkirchen, Germany). Cell debris were removed either by centrifugation twice at 4,600 g at 4°C for 2 min or by filtration with PD-10 desalting columns (GE-Healthcare, Buckinghamshire, UK). To remove residual DTT, 2.1 mL of clear lysate were transferred to 3 Vivaspin™ sample concentrators (10 MWCO, Satorius, Goettingen, Germany) for membrane ultrafiltration by centrifugation at 14,000 g for 25 min at 17°C. Supernatants were pooled and adjusted to 2 ml with sodium phosphate buffer pH 7 without DTT.

### Differential protein modification in the presence and absence of cysteine blocking reagents

In order to block cysteine residues, 400–700 μL of leaf protein extracts or recombinant TGA2 in potassium phosphate buffer pH 7 were incubated with 2–4 mM of the sulfhydryl reactive compounds N-ethylmaleimide (NEM) (Sigma-Aldrich, Taufkirchen, Germany), iodoacetamide (IAM) (Sigma-Aldrich, Taufkirchen, Germany) or methyl methanethiosulfonate MMTS (Fluka, Neu-Ulm, Germany) for 1 h at 25°C. As a control, proteins were treated with the solvent ethanol (0.05%) under the same conditions. Samples were transferred to Vivaspin™ sample concentrators (10 MWCO) and centrifuged at 14,000 g for 15–25 min at 17°C to remove the cysteine specific reagents. Supernatants were then transferred to 1.5 ml reaction tubes, and protein concentration was estimated spectrophotometrically. Concentrations were adjusted to 2–8 μg/μL in the case of total soluble protein and to 1.5–3 μg/μL in the case of His-TGA2. Samples were incubated with 110 μM PGA_1_-biotin (Cayman Chemical, Ann Arbor, MI) for 2 h at 25°C or incubated with [1-^14^C] OPDA (500,000 cpm) for 4 h at 25°C. To analyze PGA_1_-biotin modification without treatment with cysteine specific reagents, protein concentrations were directly adjusted to the concentrations described above and incubated with 110 μM PGA_1_-biotin for 2 h at 25°C. To analyze the sensitivity of modification to a range of pH values, TGA2 was first modified by PGA1-biotin, followed by a buffer exchange using gel filtration and subsequent incubation in different buffer solutions (each 0.1 M) at ambient temperature for 2 h; buffers used were sodium citrate pH 2, sodium acetate pH 4, MES, pH 6, sodium phosphate pH 7.5 and pH 12, Tris, pH 9 and sodium borate, pH 11.

### SDS-PAGE and western blot analysis

Polyacrylamide gel electrophoresis was performed under denaturing conditions. Proteins were separated using 10% acrylamide gels. After SDS-PAGE, proteins were transferred to PVDF membrane (Merck, Darmstadt, Germany) and PGA_1_-biotin labeled proteins were incubated with NeutrAvidin-HRP (Thermo Scientific, Bonn, Germany) at a dilution of 1:10,000 in TBST including 3% non-fat dried milk powder. Chemoluminescence detection was done using Immobilon^TM^ HRP substrate (Millipore, Billerica, MA) according to the manufacturer`s protocol. After signal detection, western blots were stained with Coomassie to visualize protein loading. For size determination 5 μL PageRuler Plus Prestained Protein Ladder or PageRuler Prestained Protein Ladder (Fermentas, St. Leon-Rot, Germany) was used.

For experiments with [^14^C]-labeled OPDA a 12% Precise Protein Gel (Thermo scientific, Bonn, Germany) was used, stained by Coomassie G250 (Applichem, Darmstadt, Germany) and radioactivity was detected by using a Fujix BAS 2000 system (Fuji Photo Film, Tokyo, Japan)

### RES treatment of liquid grown seedlings and expression analysis by qPCR

Seedlings of Col-0 and mutant lines were grown under conditions described above. Medium of 14 d old seedlings was removed and new MS-medium containing 75 μM OPDA or PGA_1_ was added. After 4 h of incubation, seedlings were harvested and frozen in liquid nitrogen. As negative control, medium containing methanol (1–2% and 0.8% methanol for PGA_1_ and OPDA, respectively) was added. Total RNA was extracted from 8 seedlings after homogenization of plant material. RNA was extracted with Trifast (Peqlab, Erlangen, Germany) following the manufacturer`s instructions. 1 μg of total RNA was treated with DNaseI (Invitrogen, Karlsruhe, Germany) according to the manufacturer’s protocol. cDNA synthesis was accomplished by M-MLV Reverse Transcriptase (Promega, Mannheim, Germany). qPCR analysis was performed with SYBR-Green Capillary Mix (ThermoFisher Scientific, Hamburg, Germany) and CFX96 Touch™ qPCR-machine (Bio-Rad, Munich, Germany). Primers used for qPCR are listed in Table 1.

**Table 1:** qPCR primer sequences and accession numbers.

Primer	Primer sequence (5‘-3‘) Forward Reverse	Gene locus
***Actin2/8***	GGTGATGGTGTGTCT	At3g18780/At1g49240
	ACTGAGCACAATGTTAC	
***TolB***	CAACAGACTCTATTTCATC	At4g01870
	CGCTCGCAGATAACCACTC	
***GST25***	CTCGGTGGGAAAAGTTTAG	At2g29420
	AAACATTAAGTGACAGAAC	
***CYP81D11***	ATTGCCGAGGTAGTTGT	At3g28740
	TTGCCTTTCGTAATACT	

### [1-^14^C]OPDA synthesis from [1-^14^C]linolenic acid

[1-^14^C]OPDA was enzymatically synthesized from [1-^14^C]linolenic acid 98% (Biotrend, Cologne, Germany) using a modified version of the original protocol [21]. [1-^14^C]α-linolenic acid (10 μCi) and 5 mg linseed acetone powder were dissolved in 700 μL borate buffer (1 M, pH 7.5) and incubated on an orbital shaker at room temperature for 30 min. The reaction was stopped by acidification with 100 μL 1 M citric acid. Extraction of [1-^14^C]OPDA was performed with 600 μL ethyl acetate and centrifugation at 9200 g at room temperature. The upper organic phase was transferred into a new 2 ml reaction tube. This extraction was repeated twice. The combined extract was dried and reconstituted in 100 μL acetonitrile for HPLC analysis.

HPLC was equipped with a 600E quarternary pump, a 717plus autosampler and a 996 diode array detector (Waters, Milford, USA). Separation was done on a Purospher Star RP18e column (250 × 4 mm, 5 μm particle size; with guard column, 4 × 4 mm, Merck, Darmstadt, Germany) with a linear gradient starting from 55% solvent A (water containing 0.1% acetic acid) to 52% solvent B (acetonitrile) within 28 min at a flow rate of 1 ml/min. UV absorption was monitored from 200 to 400 nm, OPDA was detected at 222 nm.

### *In vivo* labeling of proteins with [1-^14^C]OPDA

Leaf discs 0.5 cm in diameter were cut from plants (Col-0) grown in soil for 6–8 weeks. A total of 20 leaf discs were floated overnight on 2 ml deionized H_2_O in a 6-well plate in the dark at ambient temperature. Leaf discs were then exposed to 1.7 x 10^6^ cpm (46 μM) [1-^14^C]OPDA in 10 mM MES, pH 6. Labelling occurred for 2 h at ambient temperature and light conditions. Leaf discs were washed three times in a small (3.5 cm diameter) Petri dish with 10 ml H_2_O, each. Leaf discs were dried with absorbent paper and extracted in 100 mM Tris, pH 8; 5 mM EDTA; 150 mM NaCl; 10 mM DTT; plant protease inhibitor cocktail (Sigma) at a dilution of 1:200. Extracts were centrifuged for 2.5 min at 12,000 g. A 1 μl aliquot of the supernatant was measured with a scintillation counter and 10 μl of the extract was applied to a lane for SDS-PAGE.

### Labeling efficiency of His-TGA2 with [1-^14^C]OPDA

To analyze the degree of TGA2 modification by RES, His-TGA2 was purified as described above. To provide an excess of [1-^14^C] OPDA, a stoichiometric ratio of 0.125 nmol TGA2 to 3.75 nmol [1-^14^C] OPDA was chosen. TGA2 (46 μg) in 50 μl potassium-phosphate buffer pH 7.5 was incubated with [1-^14^C] OPDA for 4 h at 25°C. After RES modification, the volume was adjusted to 500 μL with sodium phosphate buffer and transferred to Vivaspin™ sample concentrators (10 MWCO) to remove unbound [1-^14^C]OPDA. Probes were centrifuged at 14,000 g for 30 min at room temperature and after centrifugation additional 500 μL were added. This step was repeated 3 times and eluates were collected. Residual TGA2 bound to [1-^14^C]OPDA supernatant was removed and volume and concentration were determined. Afterwards, remaining [1-^14^C]OPDA was eluted with 500 μL methanol from the column. Radioactivity of all fractions was determined using a scintillation counter (PerkinElmer precisely, 1450 LSC& Luminescence Counter, PerkinElmer Turku, Finland). Percent modification was calculated by the ratio of bound to unbound [1-^14^C]OPDA.

### Treatment of *GST6*::*luc* seedlings and luminescence measurement

*GST6*::*luc* seedlings (12 d-old) were treated with different RES at a final concentration of 75 μM. As a negative control, the solvent methanol was applied in the corresponding concentration (2% v/v). Seedlings were incubated for 1 h, 50 μL luciferin solution (1mM) was added and luminescence was detected with a CCD camera (VisiLuxx Imager, Visitron Systems, Puchheim, Germany) using the following measurement conditions: exposure time 10 min; binning: 4; live bin: 4; autoscale: on.

Treatment of *GST6*::*luc* seedlings with cysteine specific reagents was performed with IAM [50–500 μM], NEM [10–200 μM], MMTS [50 μM– 1 mM] and diamide [100 μM– 2 mM].

## Results

### Covalent modification of TGA2 and other plant proteins by cyclopentenones

Protein modification by RES is not well understood in plants. An *in vitro* modification assay was therefore established to analyze reactions of plant proteins with different RES species in detail. Total soluble leaf protein extract was incubated with PGA_1_-biotin, separated by SDS-PAGE and modified proteins were detected with NeutrAvidin-HRP after transfer of proteins to a polyvinylidene fluoride (PVDF) membrane. Proteins not treated with PGA_1_-biotin were also analyzed to detect plant proteins that are naturally biotinylated because these proteins are also detected by the NeutrAvidin-HRP.

**Fig 1:**
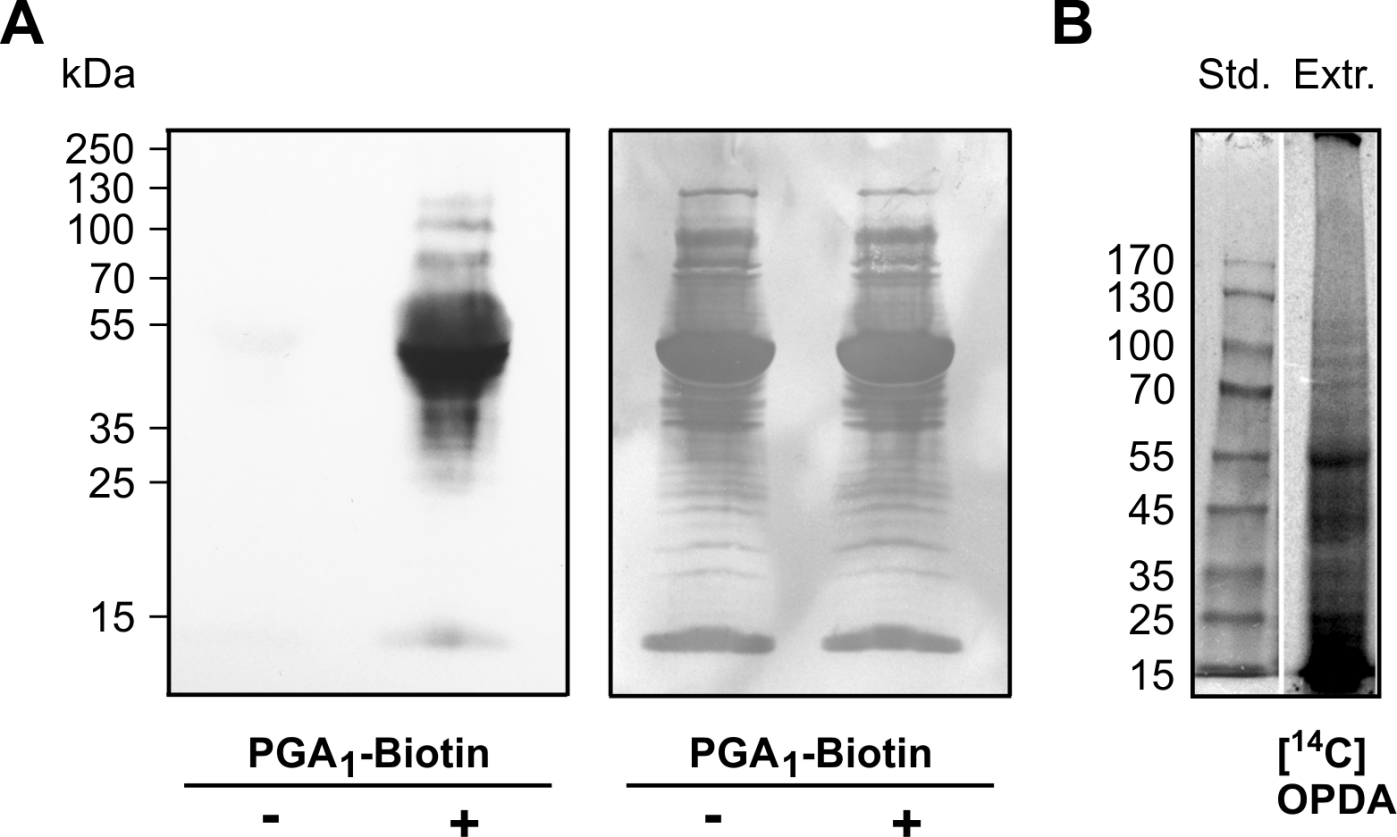
Protein modification by PGA_1_-biotin and [1-^14^C]OPDA. (**A**) Proteins were extracted from leaves and incubated with (+) or without (-) PGA_1_-biotin [110 μM]. Protein samples (120 μg) were separated on a 10% SDS-PAGE and transferred to a PVDF membrane. Biotinylated proteins were detected by NeutrAvidin-HRP (left panel) followed by protein staining with Coomassie (loading control, right panel). (**B**) Leaf discs were exposed to [1-^14^C]OPDA for 2 h. Crude extract was centrifuged and an aliquot of the supernatant was analyzed by SDS-PAGE followed by radiography.

In comparison to the untreated control, reaction of the crude protein extract with PGA_1_-biotin resulted in a much stronger response to the NeutrAvidin-HRP (Fig 1A), suggesting that few proteins are naturally biotinylated. The most abundantly labeled protein was ribulose-1,5-bisphosphate carboxylase (Rubisco); Rubisco was found to be partially biotinylated under natural (untreated) conditions. The labeling pattern shows that in addition several other proteins of different molecular sizes were modified by PGA_1_-biotin (Fig 1A) and [1-^14^C]OPDA (Fig 1B); radiolabeled OPDA was prepared from [1-^14^C]α-linolenic acid (S1 Fig). The protein modification was stable under reducing and denaturing conditions during SDS-PAGE indicating that covalent modification occurred. Endogenous *in vivo* biotinylation did not interfere with PGA_1_-biotin modification of proteins since signals of naturally biotinylated proteins were relatively weak.

### Covalent modification of TGA2 by cyclopentenones

**Fig 2:**
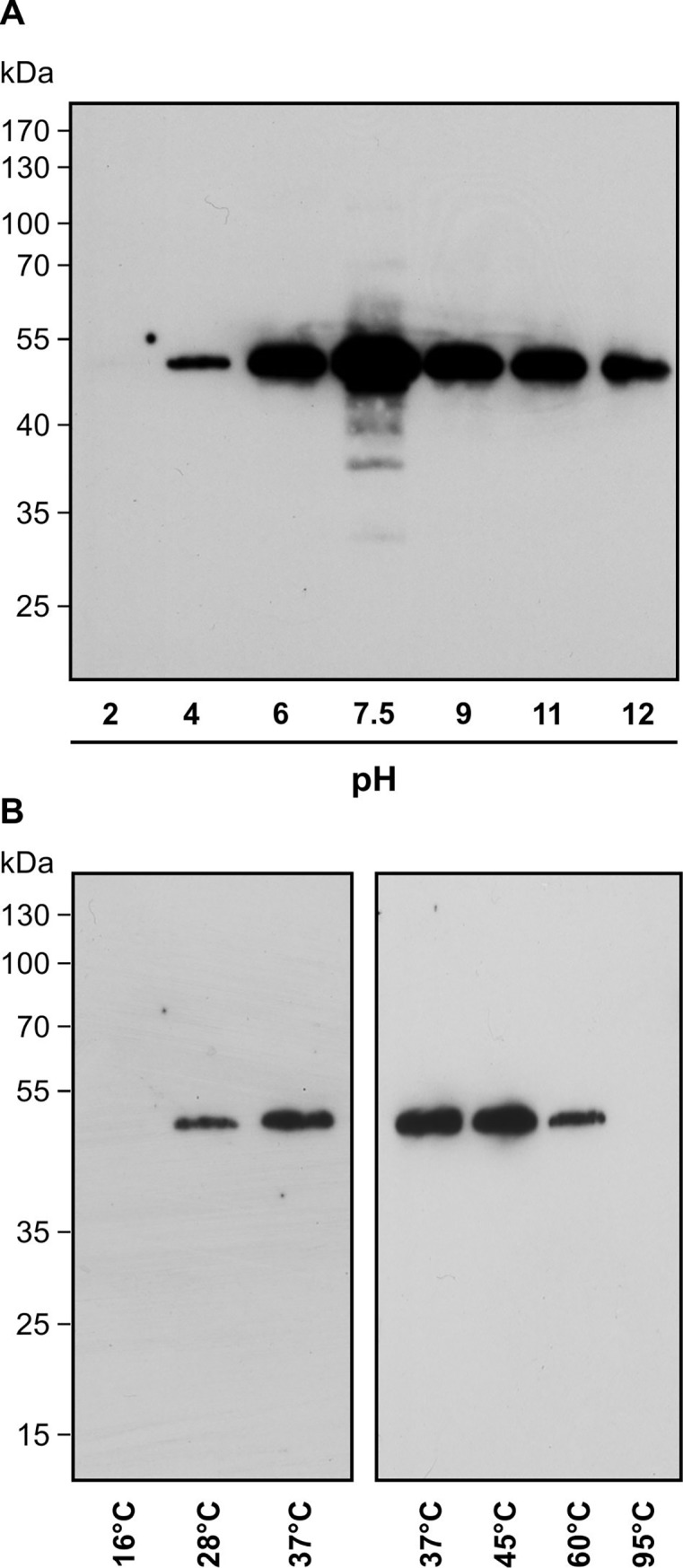
TGA2 modification by PGA_1_-biotin *in vitro*. Recombinant His-tagged TGA2 was incubated with 75 μM PGA_1_-biotin for 2 h. Proteins were separated on SDS-PAGE and transferred to a PVDF membrane. Biotinylated proteins were visualized by NeutrAvidin-HRP. **(A)** Stability of modification under different pH conditions tested at ambient temperature. **(B)** Incubation of TGA2 with PGA_1_-biotin in sodium phosphate, pH 7.5 at different temperatures. His-TGA2 has a predicted molecular weight of 42.3 kDa. The addition of six histidine residues may alter the migration slightly, resulting in a larger than expected protein band of ~50 kDa (Figs 2 and 3). Modification by PGA_1_-biotin would only add 0.6 kDa if a single site was modified. Immunoblot analysis with an αTGA2 antibody [22] confirmed the identity of His-TGA2 at 50 kDa (data not shown).

The transcription factor TGA2 contributes to cyclopentenone-induced regulation of gene expression. RES-dependent post-translational modification may play a role in controlling the activity of TGA2 because no change in expression of TGA2 upon RES-oxylipin treatment was evident [2]. To investigate whether TGA2 is covalently modified by cyclopentenones, TGA2 containing a His-Tag was expressed in *E*. *coli*, purified and tested for binding of PGA_1_-biotin. As shown, TGA2 was modified by PGA_1_-biotin (Fig 2).

Covalent modification of TGA2 was sensitive to extreme pH conditions and temperatures. Modification of TGA2 by PGA_1_-biotin was disrupted after incubation of the modified protein in acidic buffer, pH 2 (Fig 2A). In contrast, exposure of TGA2 in buffers over a range from pH 4 up to pH 12 led to a strong modification of TGA2. This suggests that covalent TGA2 modification is sensitive to acidic conditions. The temperature optimum for PGA_1_-biotin modification of TGA2 was 37°C to 45°C (Fig 2B). TGA2 was not modified by PGA_1_-biotin at 16°C and 95°C.

In addition to TGA2 modification by commercially available PGA_1_-biotin, a RES not occurring *in planta*, we examined the capacity of OPDA, an important plant RES, to modify TGA2. Since no biotinylated OPDA was available, radioactively labeled OPDA was prepared from [1-^14^C]α-linolenic acid (S1 Fig). The radioactive label additionally enabled the quantitation of OPDA bound to TGA2 from which the proportion of modified TGA2 could be calculated. Recombinant TGA2 was purified and incubated with [1-^14^C]OPDA at a stoichiometric ratio of 1:3 to guarantee an excess of the RES species. Measurement of free and TGA2-bound [1-^14^C]OPDA in three independent experiments indicated an *in vitro* modification efficiency of 3.84% ± 0.59% (mean ± SD).

### PGA_1_-biotin modifies Cys_186_ of His-TGA2 but this residue does not alter RES-induced gene expression

PGA_1_ contains a cyclopentenone ring with an α,ß-unsaturated carbonyl group, which can react with thiol groups (Cys) and/or primary amines (Lys, Arg) in proteins [10]. As thiol modification is the predominant mechanism of conjugate formation, the possibility of PGA_1_ forming stable, covalent adducts with TGA2 by Michael addition to thiols was examined.

**Fig 3:**
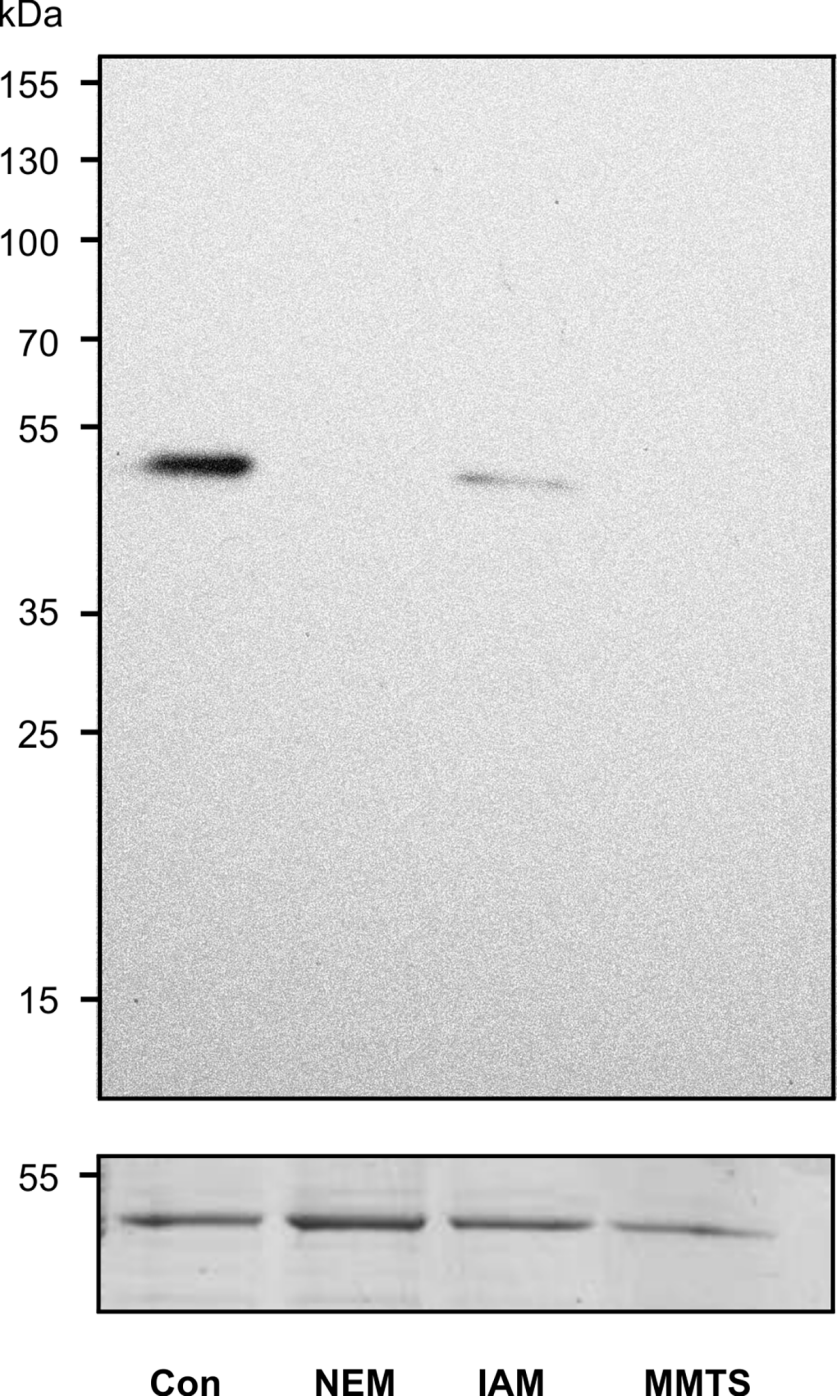
Effect of cysteine blocking reagents on TGA2 modification by PGA_1_-biotin. Recombinant TGA2 was incubated with the cysteine reactive reagents N-ethylmaleimide (NEM), iodoacetamide (IAM) or S-methyl methanethiosulfonate (MMTS) prior to PGA_1_-biotin modification. Protein samples (30 μg) were separated on SDS-PAGE and transferred to a PVDF membrane. Biotinylated proteins were visualized by NeutrAvidin-HRP, followed by protein staining with Coomassie (loading control, lower panel).

Specifically, interference of the cysteine reactive compounds N-ethylmaleimide (NEM), iodoacetamide (IAM) and S-methyl methanethiosulfonate (MMTS) with PGA_1_-biotin modification was tested. Prior to incubation with PGA_1_-biotin, these thiol blocking reagents were applied at concentrations of 2 to 4 mM to interfere with conjugation of PGA_1_-biotin to cysteine in TGA2. Blotting and detection with NeutrAvidin-HRP clearly showed that PGA_1_-biotin modification of TGA2 was abolished after blocking the thiol group (Fig 3). This result suggests that PGA_1_-biotin modifies TGA2 at Cys_186_ via Michael addition as TGA2 contains only a single cysteine residue. Therefore, elucidating cysteine modification as the modification mechanism identified this cysteine as the critical amino acid responsible for PGA_1_-binding.

**Fig 4:**
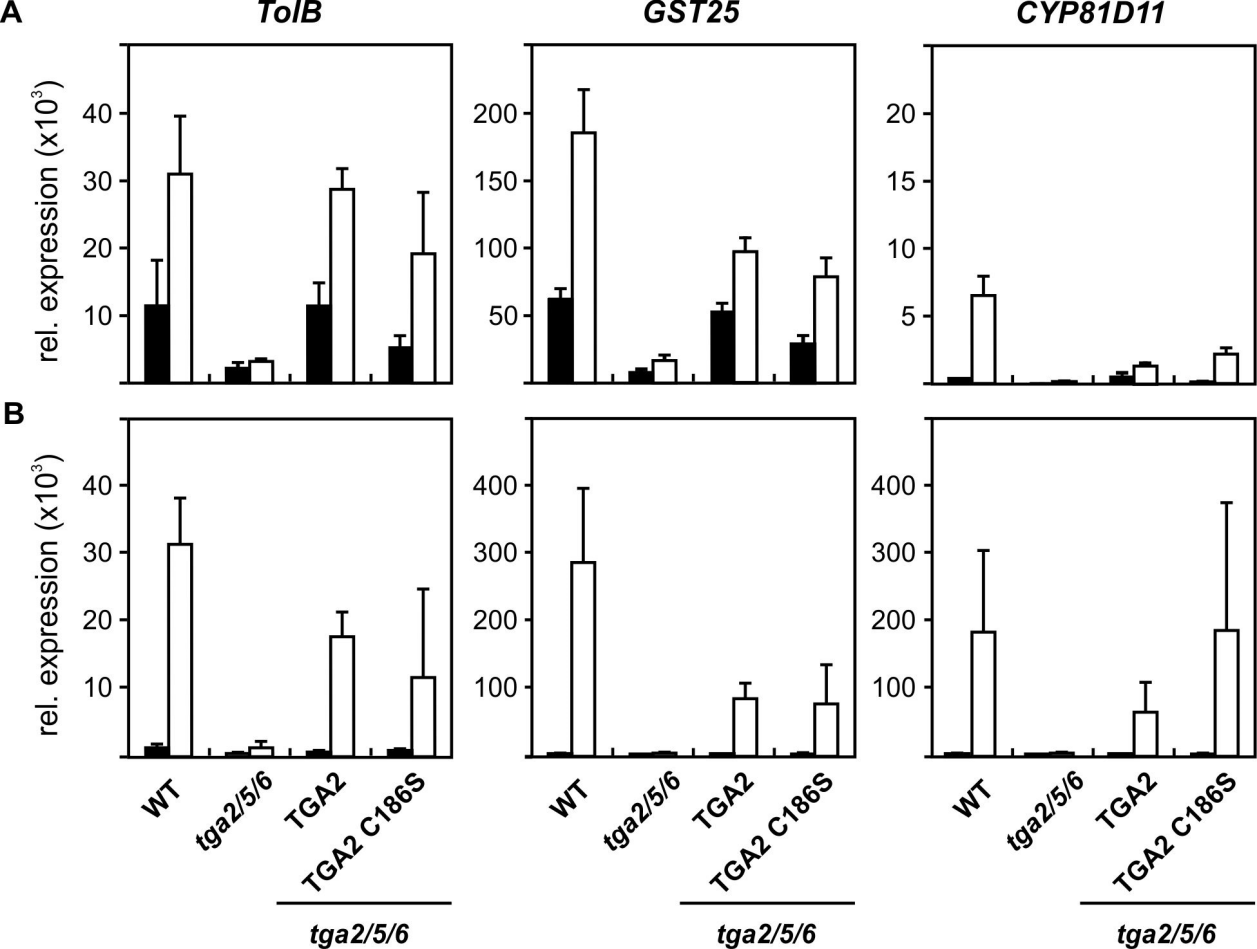
Induction of oxylipin-responsive genes by RES in wild type and TGA mutant lines. Wild type and different mutant seedlings (14 d) were incubated without (black bars) or with (white bars) 75 μM PGA1 (**A**) or 75 μM OPDA (**B**) for 4 h. The following lines were tested: *tga2/5/6* (*tga2 tga5 tga 6* triple knock-out line), TGA2 (native TGA2 overexpression in the background of *tga2/5/6*, TGA2 C186S: Overexpression of mutant TGA2 C186S in the background of *tga2/5/6*. RNA was isolated and qPCR analysis was performed. Expression of *TolB*, *GST25* and *CYP81D11* is relative to 10,000 molecules *Actin2/8*. Data show means ± SD, n = 5. Similar results were obtained in two additional experiments for PGA_1_ and another experiment for OPDA treatment with different TGA2 and TGA2_C186S_ lines.

To analyze the biological relevance of TGA2 modification by RES *in vivo*, transgenic Arabidopsis lines expressing wild type *TGA2* or a mutant with a cysteine_186_ to serine substitution (*TGA2*_*C186S*_) under the control of the *CaMV 35S* promoter in the background of a *tga256* triple knock-out mutant were generated. Treatment with PGA_1_ and OPDA was used to determine whether expression of detoxification genes is dependent on cysteine_186_ modification of TGA2 by RES. Exposure of seedlings to RES-oxylipins and subsequent qPCR analysis demonstrated strong induction of the detoxification genes *TolB*, *GST25* and *CYP81D11* in wild type plants, which was nearly abolished in the *tga256* triple knockout mutant. This is in agreement with published data and confirms the essential function of class II transcription factors for induction of gene expression in response to RES-oxylipins [2, 3]. Expression of wild type TGA2 in the *tga256* background partially restores the wild type phenotype. Partial but not complete complementation in four out of six qPCR studies with OPDA and PGA_1_ (Fig 4.) may be due to the fact that expression results from a cDNA under the control of the heterologous *CaMV 35S* promoter, which sometimes leads to less functional proteins as compared to those expressed from genomic constructs, or may be due to requirement of heterodimerization with other class II TGA factors for full induction of gene expression.

Importantly, the *tga256* triple mutants overexpressing wild type *TGA2* and the *TGA2*_*C186S*_ mutation did not differ in RES-induced gene expression (Fig 4). This shows that TGA2 modification by RES-oxylipins at cysteine_186_ is not crucial for induction of the detoxification genes *TolB*, *GST25* and *CYP81D11*.

### Modification of cysteines in other proteins by PGA_1_-biotin and [1-^14^C]OPDA

**Fig 5:**
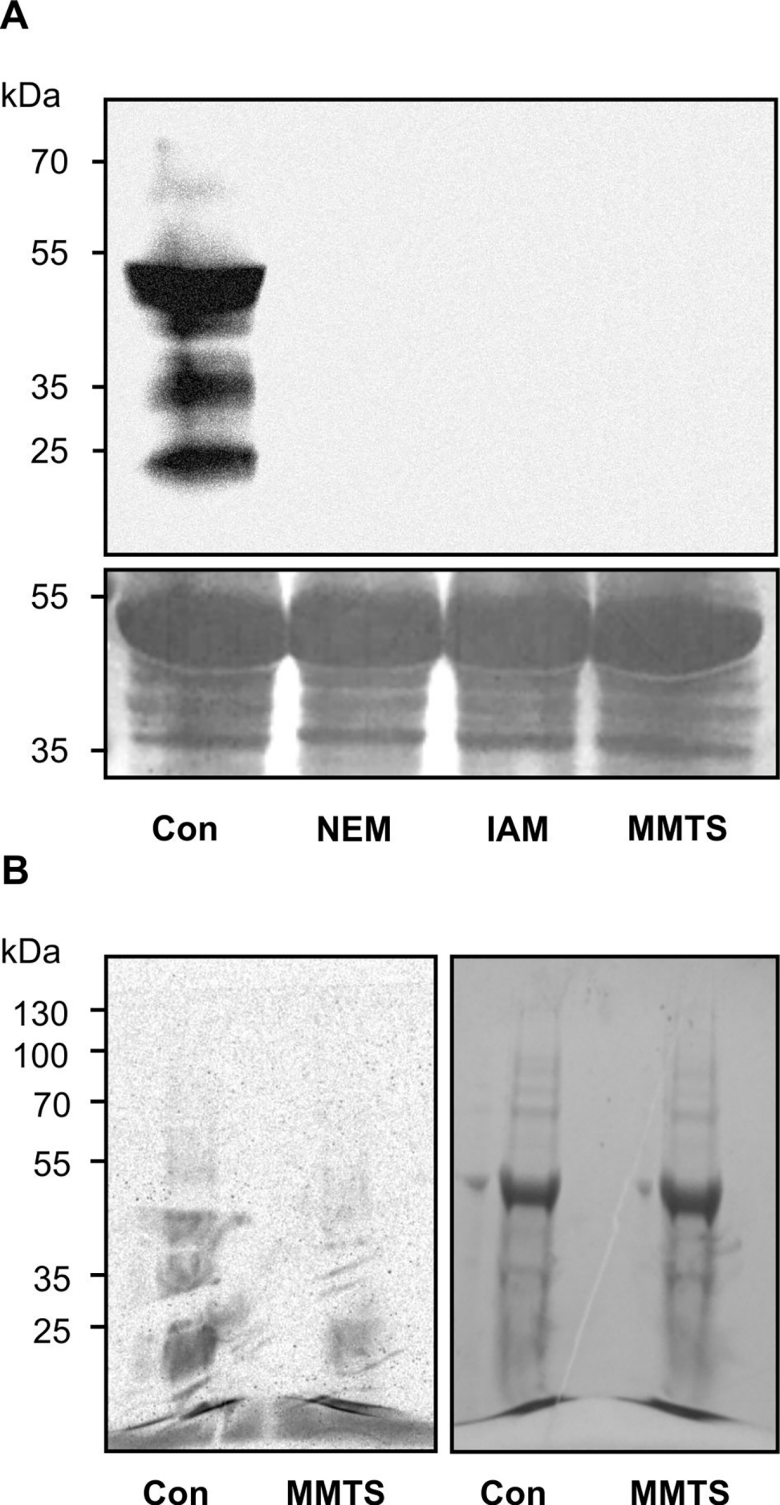
Cysteine specific modification of proteins by RES. Proteins were extracted from leaves and incubated without (control, Con) or with the cysteine reactive reagents N-ethylmaleimide (NEM), iodoacetamide (IAM), S-methyl methanethiosulfonate (MMTS) for 1 h prior to RES lipid modification. Protein samples were separated by SDS-PAGE. (**A**) After modification with PGA_1_-biotin and SDS-PAGE, proteins (90 μg) were transferred to a PVDF membrane. Biotinylated proteins were visualized by NeutrAvidin-HRP (upper panel) followed by protein staining using Coomassie blue (loading control, bottom panel). (**B**) After modification by [1-^14^C]OPDA (500000 cpm) for 4 h, the SDS–PAGE gel (loaded with 25 μg protein per lane) was analyzed by autoradiography (left panel) and Coomassie staining (right panel).

Although TGA2 modification by PGA_1_-biotin does not affect the biological function of TGA2, modification of other proteins by RES may contribute to TGA2-mediated signaling. Therefore total protein modification by PGA_1_-biotin and [1-^14^C]OPDA was analyzed. Soluble proteins were extracted from wild type plants and incubated with the cysteine reactive reagents NEM, IAM and MMTS prior to modification by PGA_1_-biotin or [1-^14^C]OPDA. Western blot analysis (Fig 5A) of PGA_1_-biotin modified crude extracts clearly indicated that all proteins were modified at cysteine residues, because pretreatment with thiol blocking reagents abolished PGA_1_-biotin modification. This result showed that, similarly to TGA2, PGA_1_-biotin exclusively modified proteins at cysteine residues. In contrast, modification of proteins by [1-^14^C]OPDA was not completely abolished but diminished by pretreatment with MMTS (Fig 5B). Thus, [1-^14^C]OPDA also modified several proteins at cysteine residues. However, it could not be excluded that other amino acid residues (like primary amines) were also targeted by covalent [1-^14^C]OPDA binding.

### Analysis of the biological activity of different RES-oxylipins

**Fig 6:**
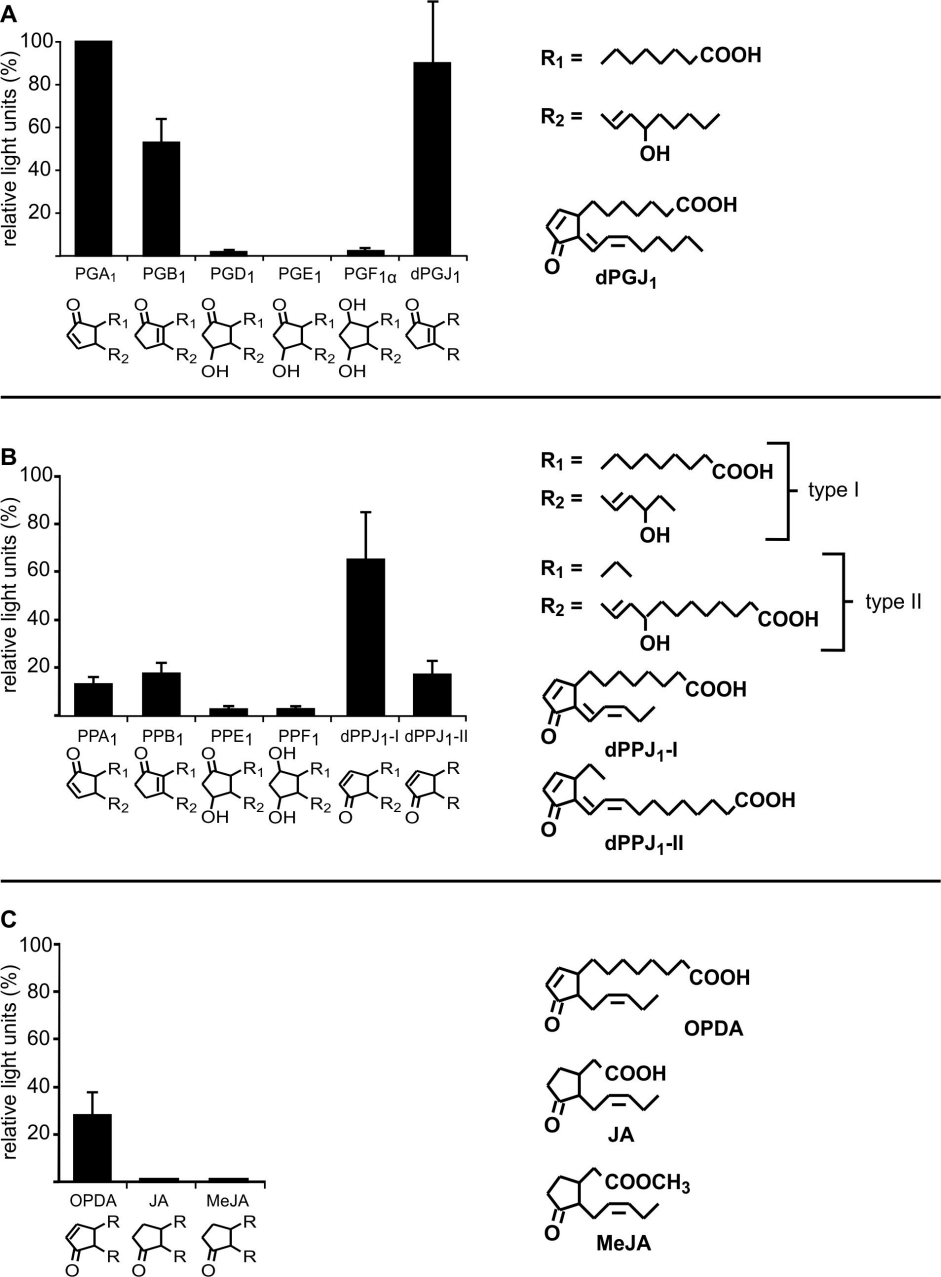
*GST6*::*luc* activation by different oxylipins. *GST6*::*luc* seedlings (10 d old) grown in 24 well plates in liquid MS media were incubated with different prostaglandins **(A)**, phytoprostanes **(B)** or jasmonates **(C)** at a final concentration of 75 μM. All phytoprostanes were racemic mixtures of type I and II regioisomers except for dPPJ_1_-I and dPPJ_1_-II. Stereochemistry is not indicated. Luminescence was measured after 7 h of treatment. In all experiments, luminescence after PGA_1_ treatment was set to 100% and relative light units of all other treatments were calculated. Bars indicate relative light units of three independent samples (means ± SD).

In order to analyze which properties of reactive electrophilic oxylipins are essential for gene induction, experiments with *GST6*::*luc* plants were performed. GSTs are induced by different abiotic and biotic stresses like heavy metals and pathogens but also by ROS and RES [23–25]. Therefore the noninvasive *GST6*::*luc* system is suitable for rapid screening of the biological activity of different RES-oxylipins including prostaglandins (Fig 6A), phytoprostanes (Fig 6B), and jasmonates (Fig 6C).

Transgenic *GST6*::*luc* seedlings were exposed to oxylipins with varying cylopentane-ring systems (Fig 6). Of the oxylipins tested, PGA_1_ was the strongest inducer. Relative light units after treatment with each of the tested oxylipins were therefore expressed relative to PGA_1_-induced luciferase activity set to 100% (Fig 6). All cyclopentenones (PGA_1_, PGB_1_, dPGJ_2_, PPA_1_, PPB_1_, dPPJ_1_ and OPDA) induced luciferase activity albeit to varying degrees. Relative to PGA_1_ induced luciferase activity, dPGJ_2_ and dPPJ_1_ were with 85% and 65% the strongest inducers, whereas induction after OPDA treatment was lower with 28%. By comparison, induction of luminescence by the tested cyclopentanones (PGD_1_, PGE_1_, PGF_1α_, PPE_1_, PPF_1_, JA and MeJA) was very low (<5%). These results demonstrate that oxylipins require the cysteine-reactive α,β-unsaturated carbonyl group to induce *GST6* expression. Notably, the side chains attached to the cyclopentenone ring system also influence the biological activity of the oxylipins. For instance, treatment of seedlings with the regioisomer dPPJ_1_-I resulted in 65% of the PGA_1_-induced promoter activity, whereas treatment with dPPJ_1_-II only induced 20% of *GST6*::*luc* activity relative to PGA_1_ (Fig 6).

### Impact of cysteine-reactive reagents on *GST6* promoter activity

**Fig 7:**
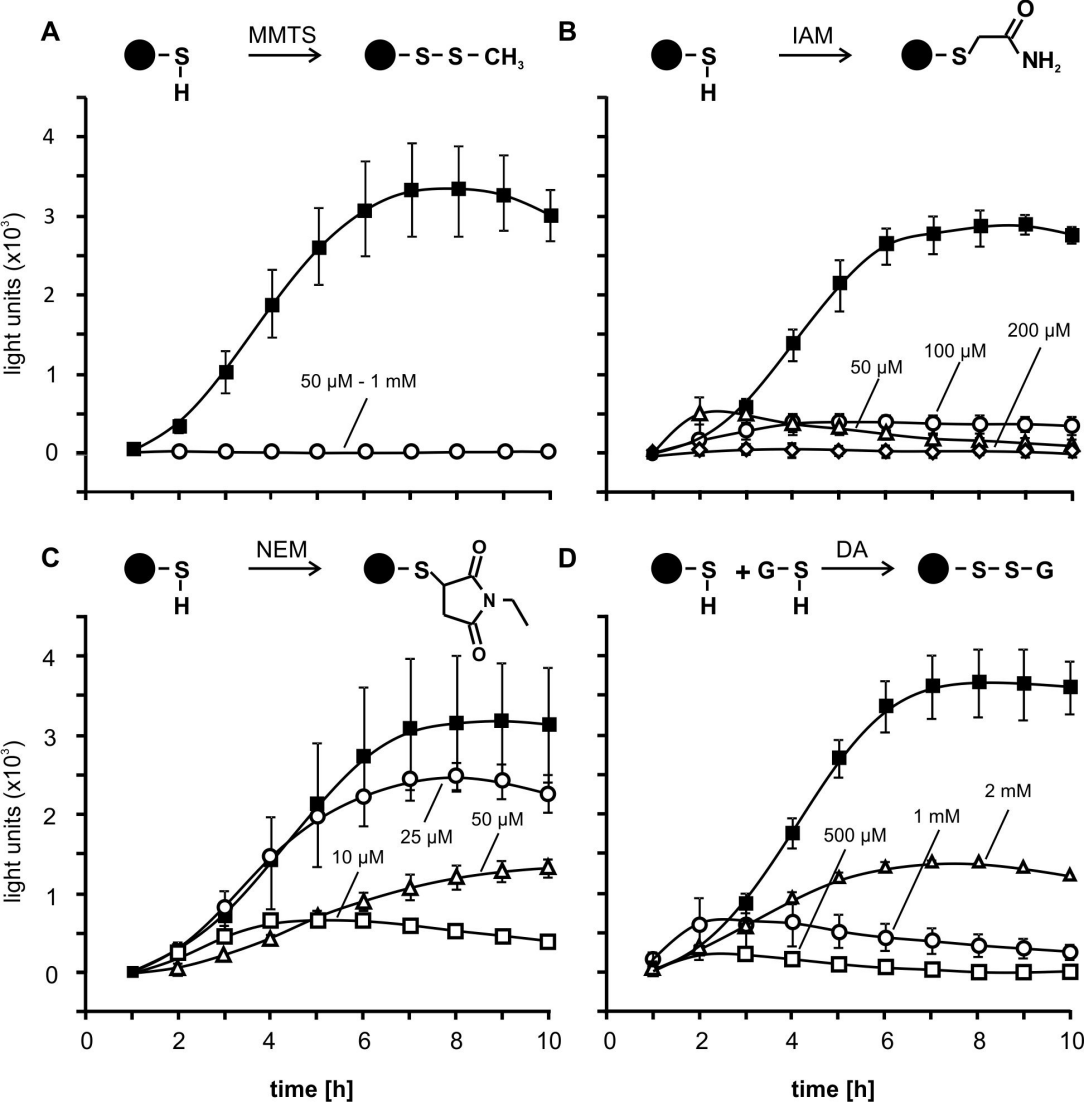
*GST6*::*luc* promoter activity after treatment with thiol specific reagents. *GST6*::*luc* seedlings grown in liquid MS medium (10 d old) were incubated with the thiol specific reagents (**A**) S-methyl methanethiosulfonate (MMTS), (**B**) iodoacetamide (IAM), (**C**) N-ethylmaleimide NEM and (**D**) diamide at different concentrations as indicated. Luminescence was measured 1–10 h after treatment. Symbols indicate mean values of three independent replicates ± SD. For comparison, black squares show the response to treatment with PGA_1_ (75 μM). Cartoons of reactions between proteins (black circles) and thiol-specific reagents are shown above the corresponding charts.

The results obtained in this study suggest that cyclopentenone oxylipins bind to different proteins or transcription factors and induce gene expression of detoxification genes like *GST6*. To further investigate structure-activity relationships we tested structurally different thiol-modifying reagents using the *GST6*::*luc* line (Fig 7). Application of MMTS, which blocks cysteines via S-thio-methylation (Fig 7A) and IAM, which covalently modifies the thiolate anion by SN_2_ displacement (Fig 7B) did not or only weakly induce *GST6*::*luc* activity. In contrast, NEM which covalently binds to cysteine residues by Michael addition induced *GST6* promoter activity in a concentration dependent manner. Strongest induction was obtained by application of 25 μM NEM while higher concentrations resulted in a decrease of promoter activity that could be the result of toxic effects (Fig 7C).

We also tested diamide, a reagent which leads to glutathionylation of thiols of proteins and to the formation of oxidized glutathione (GSSG) by glutathionylation of glutathione (GSH). The latter reaction changes the cellular redox potential which could induce redox-regulated genes independent of protein modification. Low diamide concentrations (500 μM and 1 mM) exerted no effect on *GST6* promoter activity, but 2 mM activated the promoter (Fig 7D). These results suggest that conjugation of proteins with GSH or changes of the redox potential also induces gene expression albeit to a much lower extent than modification by PGA_1_. Moreover, a substantially higher concentration of diamide was needed to induce *GST6* expression relative to PGA_1_ and NEM.

## Discussion

In animals, covalent modification of thiol groups of proteins is an established signalling mechanism [12]. One important example is the recognition of lipophilic RES substances such as xenobiotics in animals by modification of specific cysteine residues of KEAP1 which regulates the interaction with the transcription factor NRF2 [26]. Because of the reactivity of RES-oxylipins, thiol modification might be involved in RES-oxylipin signalling in plants as well. Here, thiol modification of plant proteins was investigated by analyzing the pattern of proteins modified by PGA_1_ or OPDA in combination with thiol-modifying reagents and by studying the impact on the regulation of gene expression.

### Specificity of thiol modification in plants

One factor determining the efficiency of modification is the reactivity of the RES-compound. There are several indications that the α,ß-unsaturated carbonyl structure which confers the chemical reactivity towards thiols is important for the biological activity [2, 27–29]. Here, we systematically analysed the activity of a variety of cyclic oxylipin compounds with or without RES properties and of thiol modifying reagents as inducers of the *GST6* promoter using a reporter line. In accordance with other test systems, electrophilic cyclopentenones were generally more active than non-RES cyclopentanones (Fig 6). *In vivo*, the ability of different RES to passively diffuse through membranes is essential for intracellular target protein modification. However, all tested RES and thiol reagents are membrane permeable, and, hence, differences in cellular uptake likely do not explain the differences in biological activity.

For thiol modification to function in signal transduction, RES are expected to react with thiols of RES-sensing proteins much faster than with the bulk of proteins and glutathione. Due to the high reactivity of RES, a subset of cellular proteins was modified by RES as can be seen from the pattern in Figs 1 and 5. Although cysteine residues are present in the majority of proteins, different proteins are apparently modified with different reaction velocities. What are the reasons for preferential modification of specific proteins? Firstly, cysteines that can be modified by RES are typically located on the protein surface, accessible to modifying substances and should not be buried within the protein [30]. Secondly, the microenvironment in the protein strongly determines the reactivity of protein thiols. Notably, the reaction of RES and thiol-reactive reagents with cysteine residues in their neutral form is slow. In contrast, the deprotonated thiolate form of a cysteine is a strong nucleophile and reacts rapidly with RES and thiol reagents. Therefore, the cysteine is more reactive when the neighboring amino acids support the formation of thiolates, i.e. decrease the pK_a_ value [30]. Thirdly, RES displaying affinity to protein target sites will react faster with thiols at targets sites than RES and thiol blockers with low affinity.

**Fig 8:**
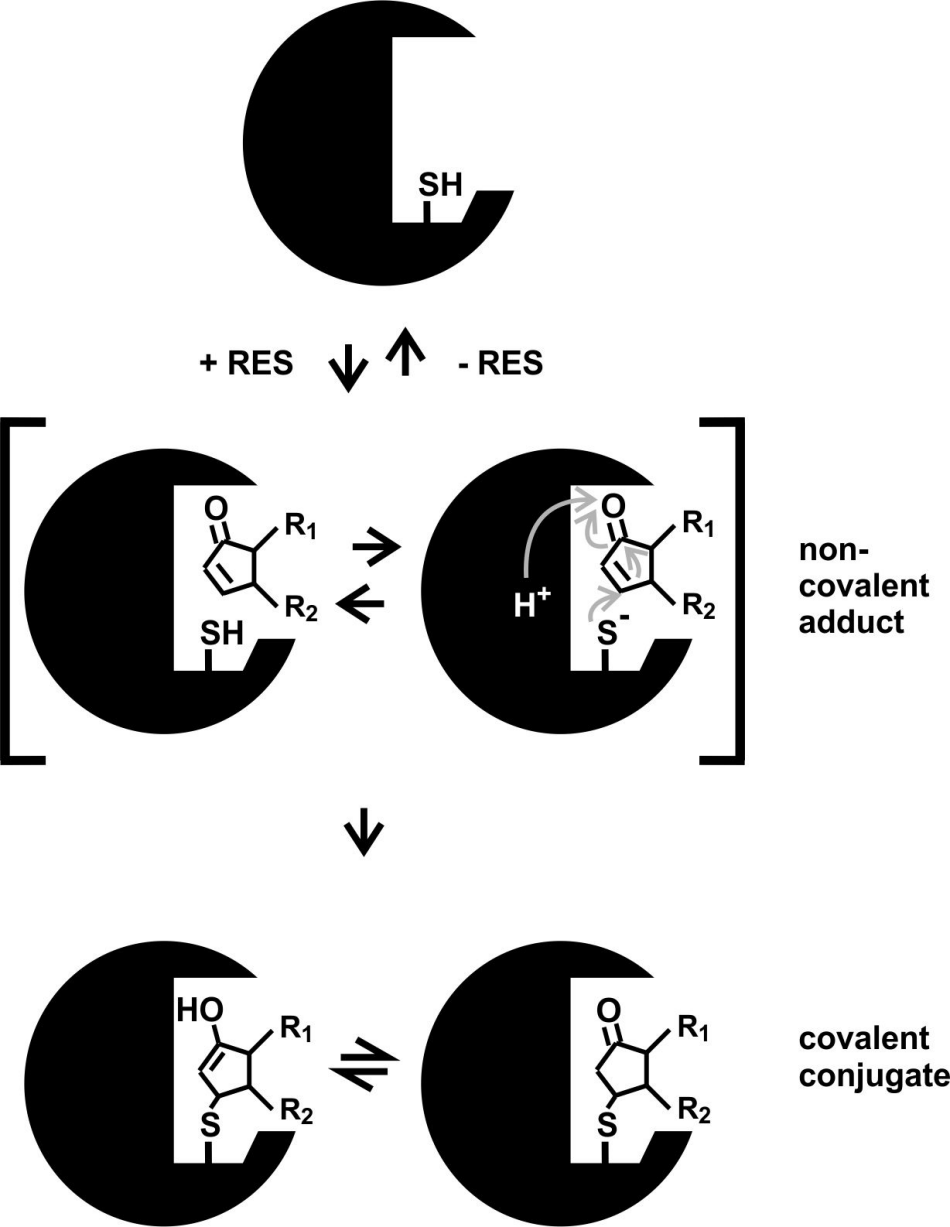
Proposed mechanism of RES/cyclopentenone modification at cysteine residues of proteins. The α,ß-unsaturated carbonyl group of RES acts as an electrophile and modifies the thiol group of proteins *via* Michael addition. In a first reversible step, a non-covalent adduct of a protein with a RES may occur thereby facilitating the subsequent irreversible reaction of an exposed thiolate with the α,ß-unsaturated carbonyl group of the RES leading to covalent conjugate formation. Changes of the target protein conformation by non-covalent and covalent ligand binding may lead to target activation or inhibition.

When comparing different RES and thiol reactive reagents (Figs 6 and 7) with respect to their *GST6* promoter inducing activity, it is apparent that thiol reactivity appears to be important but not sufficient for biological activity. Results suggest that structural properties other than thiol-reactivity are critical. For instance, some thiol-reactive molecules may not have access to thiols in protein pockets due to steric reasons. This is likely not a problem for small thiol reagents like MMTS or IAM that are often used to block thiols in proteins. Notably, the reaction velocity of thiol reactive molecules with proteins is high when the compound displays an affinity to target proteins, i.e. binds non-covalently to the protein surface close to exposed cysteine thiolates. Non-covalent binding of structurally different thiol reactive molecules prior to covalent thiol modification is expected to be strongly dependent on the steric properties and molecular interactions between the RES or thiol reagent and a target protein (Fig 8). After binding of biologically active ligands to their protein target sites, the ligand is thought to induce a conformational change in the target protein resulting in its inhibition or activation. Biologically active RES may differ in their affinity to targets sites and also differ in their ability to alter protein conformation. Small thiol-reactive reagents unable to activate the *GST6* promoter such as MMTS and IAM may covalently bind to exposed protein thiols but lack the capacity to induce functionally relevant conformationally changes.

It should be noted that non-covalent binding of the ligand to target protein sites could be sufficient to induce a conformational change in proteins (Fig 8). Likely, many of the structurally diverse biologically active RES (and potentially structurally related inactive molecules) display low affinity to target protein sites. However, covalent ligand binding dramatically increases the stability of the ligand-protein complex and would also prevent rapid metabolism of the bound ligand. Hence, covalent binding of biologically active ligands that display low target protein affinity would dramatically lower the concentration required to activate or inhibit its target.

Hence, the rate of thiol modification at specific sites is not only dependent on the reactivity of the electrophilic group but also on the total structure of RES.

### Impact of TGA2 thiol modification on RES-oxylipin signalling

Cyclopentenones, including OPDA, are important regulators of development as well as biotic and abiotic defence responses [29, 31–33]. These RES-oxylipins were shown to activate the expression of detoxification genes in a class II TGA factor-dependent manner [2]. The mechanism by which this occurs remains to be explored ([10], see introduction). We tested the possibility that covalent modification of the transcription factor TGA2 is a signalling mechanism of RES-oxylipins in plants. Interestingly, these TGA factors also control salicylic acid- and xenobiotic-dependent gene expression as well as expression of JA/ethylene-induced defense genes [6–8, 34]. Different posttranslational modifications of these transcription factors would enable, on one hand, a fast regulation of these proteins and, on the other hand, to discriminate between the activities upon oxylipin, salicylic acid or JA/ethylene treatment. Phosphorylation of TGA factors and particularly TGA2 after salicylic acid treatment has been described before [35, 36], but the physiological relevance has not yet been proven. The same holds for the SA-induced redox-modification of TGA1 [37]. Instead, recruitment of regulated transcriptional co-activators by TGA factors is an established regulatory mechanism that controls TGA function. In the context of salicylic acid-induced gene expression, the redox-controlled TGA-interacting protein NPR1 (NON EXPRESSOR OF PR GENES1) is required for regulated gene expression [38, 39]. For xenobiotic-induced gene expression, the TGA-interacting protein SCL14 is required [8]. Whether or how SCL14 activity is controlled, has remained unknown. With respect to the essential role of class II TGA factors in JA/ethylene-induced gene expression, it has been proposed that TGA2 as such is not regulated but is rather required to enhance EIN3 (ETHYLENE INSENSITIVE3)-regulated promoter activity [40]. With respect to RES-induced gene expression, it remains to be elucidated, whether TGA2 is modified or whether it interacts with a RES-regulated protein or whether it just amplifies the activity of a yet unknown RES-responsive transcription factor.

Here, thiol modification of TGA2 by lipids with α,ß-unsaturated carbonyls was shown (Fig 2). Use of thiol-blocking reagents demonstrated that modification of TGA2 primarily occurred at a cysteine residue (Fig 3). In *A*. *thaliana*, a cysteine is required for the activity of the floral transcription factor PERIANTHIA (TGA8), as overexpression of a mutated version of this protein where cysteine_340_ was replaced by serine, was not able to restore the wild type phenotype [41]. TGA factors thus seem to contain crucial cysteine residues, which are important for the activity of these transcription factors. Likewise, TGA1 contains reactive cysteines that are reduced in SA-treated plants [42] or nitrosylated *in vitro* [37]. In the case of TGA2, however, modification of the only cysteine residue was not necessary for function in activating the expression of the cyclopentenone-responsive genes *TolB*, *GST25* and *Cyp81D11* (Fig 4). In a similar experimental set up, i.e. complementation of the *tga256* mutant with a *35S*:*TGA5* and a *35S*:*TGA5*_*C186S*_ construct, it was shown that the single cysteine of TGA5 was not important for TGA5 to induce the detoxification response after treatment of plants with the toxic chemical TIBA (2,3,5-triiodobenzoic acid) [43]. Therefore, other mechanisms such as binding of interacting proteins may be more important for regulating TGA2 activity.

Nevertheless, protein thiol modification of one or more target proteins in the TGA-signaling pathway appears to be important since a series of reactive cyclopentenones and the thiol reagent NEM activated the *GST6* promoter. In the future, identification of target proteins for RES-oxylipin binding is required to firmly establish the role of protein modification in RES-oxylipin signalling.

## Supporting information

S1 Fig Thin layer chromatography of [1-^14^C]OPDA synthesized from [1-^14^C]linolenic acid. A silica gel on TLC aluminium foil was used with a mobile phase of hexane:diethyl ether (2:1) containing 0.1% acetic acid. The TLC plate was analysed by radiography. Migration of OPDA and linolenic acid (LA) was confirmed by used of authentic standards and staining with iodine.
(TIF)
