# Supplementary figures and images for: TGA2 signaling in response to reactive electrophile species is not dependent on cysteine modification of TGA2
Source: PLoS One. 2018 Apr 2;13(4):e0195398. doi: 10.1371/journal.pone.0195398 (PMC5880405; doi:10.1371/journal.pone.0195398)

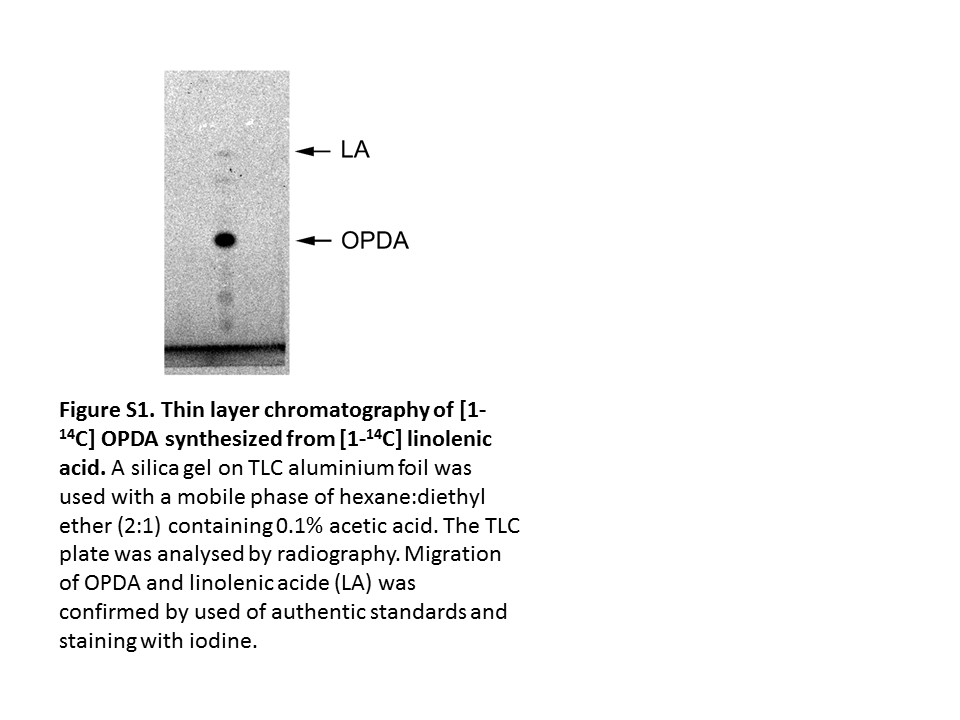

Supplement: S1 Fig — A silica gel on TLC aluminium foil was used with a mobile phase of hexane:diethyl ether (2:1) containing 0.1% acetic acid. The TLC plate was analysed by radiography. Migration of OPDA and linolenic acid (LA) was confirmed by used of authentic standards and staining with iodine. (TIF) [file pone.0195398.s001.tif]
